# Dynamic and Modularized MicroRNA Regulation and Its Implication in Human Cancers

**DOI:** 10.1038/s41598-017-13470-5

**Published:** 2017-10-17

**Authors:** Jiang Shu, Bruno Vieira Resende e Silva, Tian Gao, Zheng Xu, Juan Cui

**Affiliations:** 1 Systems Biology and Biomedical Informatics (SBBI) Laboratory, Department of Computer Science and Engineering, Lincoln, NE 68588 USA; 2 Department of Statistics, University of Nebraska-Lincoln, Lincoln, NE 68588 USA; 3 Quantitative Life Sciences Initiative, University of Nebraska-Lincoln, Lincoln, NE 68588 USA

## Abstract

MicroRNA is responsible for the fine-tuning of fundamental cellular activities and human disease development. The altered availability of microRNAs, target mRNAs, and other types of endogenous RNAs competing for microRNA interactions reflects the dynamic and conditional property of microRNA-mediated gene regulation that remains under-investigated. Here we propose a new integrative method to study this dynamic process by considering both competing and cooperative mechanisms and identifying functional modules where different microRNAs co-regulate the same functional process. Specifically, a new pipeline was built based on a meta-Lasso regression model and the proof-of-concept study was performed using a large-scale genomic dataset from ~4,200 patients with 9 cancer types. In the analysis, 10,726 microRNA-mRNA interactions were identified to be associated with a specific stage and/or type of cancer, which demonstrated the dynamic and conditional miRNA regulation during cancer progression. On the other hands, we detected 4,134 regulatory modules that exhibit high fidelity of microRNA function through selective microRNA-mRNA binding and modulation. For example, miR-18a-3p, −320a, −193b-3p, and −92b-3p co-regulate the glycolysis/gluconeogenesis and focal adhesion in cancers of kidney, liver, lung, and uterus. Furthermore, several new insights into dynamic microRNA regulation in cancers have been discovered in this study.

## Introduction

Mature microRNAs (miRNAs) are typically 20–25 nucleotides long. They hybridize with complementary sequences in the 3′-untranslated regions (UTRs) in messenger RNAs (mRNAs) and silence protein-coding genes through destabilizing mRNA or preventing translation of mRNA^1^. In humans, it was estimated that 2,588 miRNAs regulate over 60% of human genes and participate in every aspect of cellular activities in cell growth and cell death^2^. During the past decade, numerous studies have disclosed the regulatory roles of miRNAs in both the fundamental cellular processes^3–9^ and the development of complex human diseases such as cancer^10–14^.

Functional study of miRNA largely depends on the reliable identification of miRNA-mRNA regulatory interactions. Most state-of-the-art computational prediction methods, such as miRanda^15^, RNA22^16^, DIANA-microT^17^ and Targetscan^18^, are focused on primary search for nucleotide sequences complementary to the miRNA seed region (2^nd^–8^th^ bases on the 5′ end)^18^ and suffer from high false prediction because of the dramatic complexity in miRNA binding^19,20^. Recent sequencing-based interactome data provide different views in this issue. For examples, through the crosslinking, ligation, and sequencing of hybrids (CLASH) analysis^21^, 18,514 miRNA-mRNA interactions were detected and only ~22% were associated with seed region, via either contiguous or gapped complementary RNA base pairing. The same study also unveiled that ~60% of the binding sites are within coding region of mRNA, as opposed to the 3′UTR centric search by the existing algorithms. Similar findings were observed using the covalent ligation of endogenous Argonaute–bound RNAs (CLEAR)-CLIP in human hepatoma (Huh7.5) cells, where ~26% of the interactions are seed-associated and ~57% are non-3′UTR interactions^22^. In addition, compelling evidence also shows the stochastic nature of miRNA-mRNA interactions that 1) multiple miRNAs can bind to the same mRNA sequence or different copies of the same transcript - combinatorial interactions^23–26^ and 2) multiple different mRNAs, possibly along with other long non-coding RNAs and circular RNAs^27–29^, can compete for binding to the same miRNA - competitive interactions^23,30–32^, which is very similar to transcription factor (TF) regulation^33,34^. Other factors, including genetic mutations^35–38^, the competition with other RNA binding proteins^39,40^ and the conditional expression of miRNA and mRNA^41^ can also affect the status of miRNA-mRNA interactions. Each of these mechanisms stresses the dynamic miRNA regulation from a different perspective and many of them are conditional (e.g., associated with certain phenotypes or development stages). It is notable the conditional information can be captured by genomic data collected under associated conditions. However, to the best of our knowledge, currently there is no systematic assessment available on the *dynamic* miRNA regulation that assesses the changes across conditions. The first such attempt, reported in^24^, only briefly touched the combinatorial module that a set of miRNA can regulate a common set of targets in a specific condition, which hinders the practical use as a dynamic system.

We notice that the actual challenges in tackling dynamic miRNA regulation involve several major trends. First, while the reliable stratification of gene regulation networks has been lagging, the associations between miRNAs and TFs were reported in different scenarios^26,42–44^. For example, one TF gene, CTCF, controls the expression of HOXC5, which is the host gene of miR-615-3p^45,46^ while miR-615-3p interacts with CTCF transcript in CALSH experiment^21^. This type of bi-directed regulation between TF and miRNA may introduce the feedback loops to the whole gene regulation network. Likewise, in order to determine the miRNA-mediated regulation on gene expression, one should also consider other major gene regulatory factors, such as copy number variation (CNV) and DNA methylation^47^. Secondly, evidence also shows that multiple miRNAs can regulate a set of functionally relevant genes instead of the same gene in a cooperative manner, e.g., miR-17-5p, -18a-5p, -19a-3p, and -92a-3p co-regulate 44 functionally related genes, such as CCND2, TNRC6B, and PHF12^48^. Although our knowledge of cooperative miRNA regulation is extremely limited, it is envisioned that the mechanisms underlying TF non-complex co-regulation^49,50^ can be adopted in miRNA study.

In this study, we propose a new method to identify dynamic miRNA-gene interactions, particularly focusing on the conditional interactions that are associated with tumor progression, based on the integration of heterogeneous genomic information on multiple human cancers. Other gene expression regulators such as TF, CNV, and DNA methylation have also been considered when determining miRNA-mediated gene regulatory networks in each developmental stage of cancer. Moreover, we demonstrated the discovery of modularized miRNA regulation based on the identified miRNA-gene interactions.

## Results

**Figure 1:**
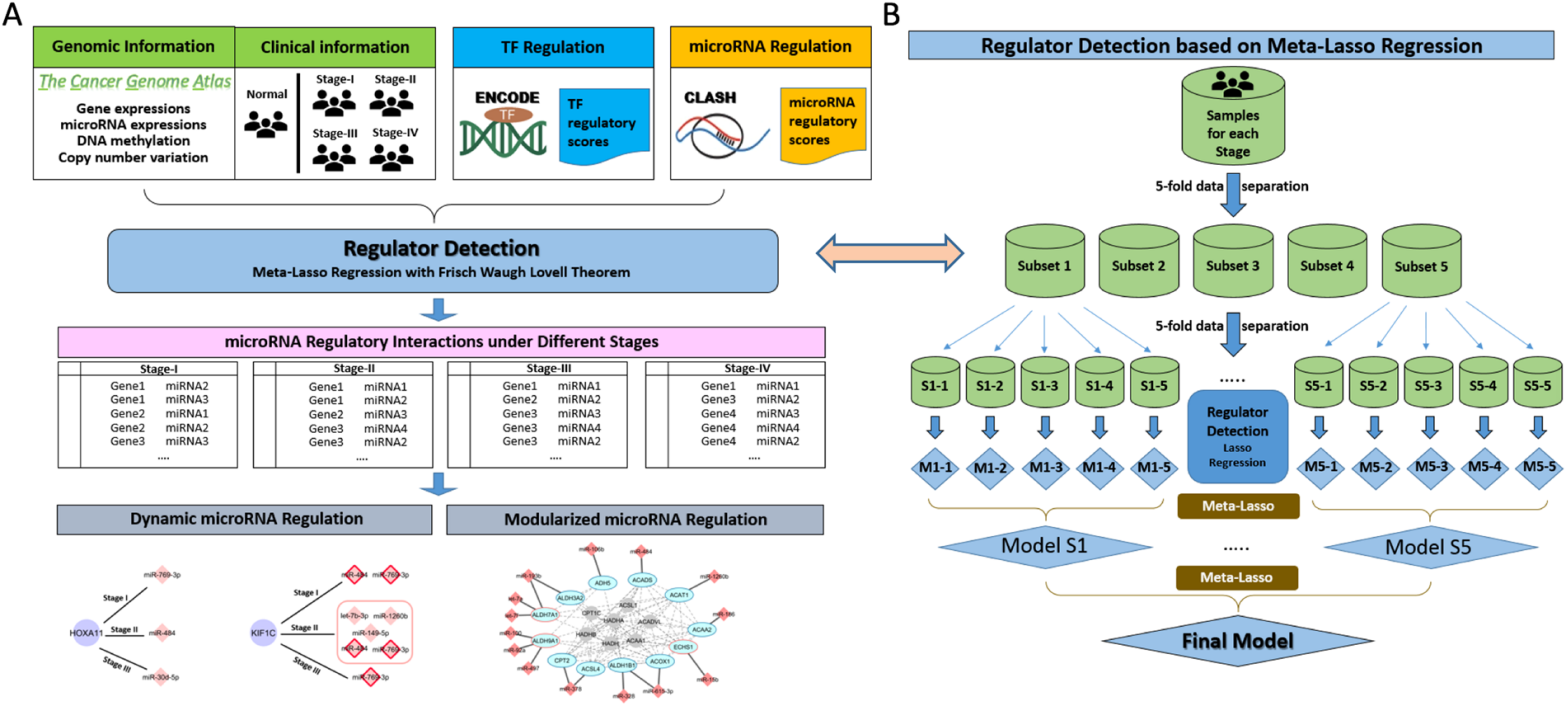
Discovery of dynamic and modularized miRNA regulation during cancer progression. (**A**) The identification pipeline of conditional miRNA regulatory interactions; (**B**) Meta-Lasso Regression approach to the detection of microRNA regulators of each gene in each cancer stage.

The workflow of this study is demonstrated in Fig. 1 with the detailed methodologies explained in Methods. We applied the proposed pipeline on cancers from seven tissues including lung, kidney, stomach, breast, liver, uterine, and pancreas, and have identified miRNA-mRNA interactions associated with each specific cancer. Note that the interactions were identified in the regulator detection step (Fig. 1B), which was performed for each cancer and each stage. While the complete results were archived in our online database (http://sbbi.unl.edu/miRDR), main discoveries were presented in the following sections. Particularly, we focused on the kidney and lung cancers and compare our discoveries between two well-defined subtypes, i.e., Kidney Renal Clear Cell Carcinoma (KIRC) versus Kidney Renal Papillary Cell Carcinoma (KIRP), and Lung Adenocarcinoma (LUAD) versus Lung Squamous Cell Carcinoma (LUSC).

### Transcript availability and binding affinity are not the only factors in miRNA regulation

**Figure 2:**
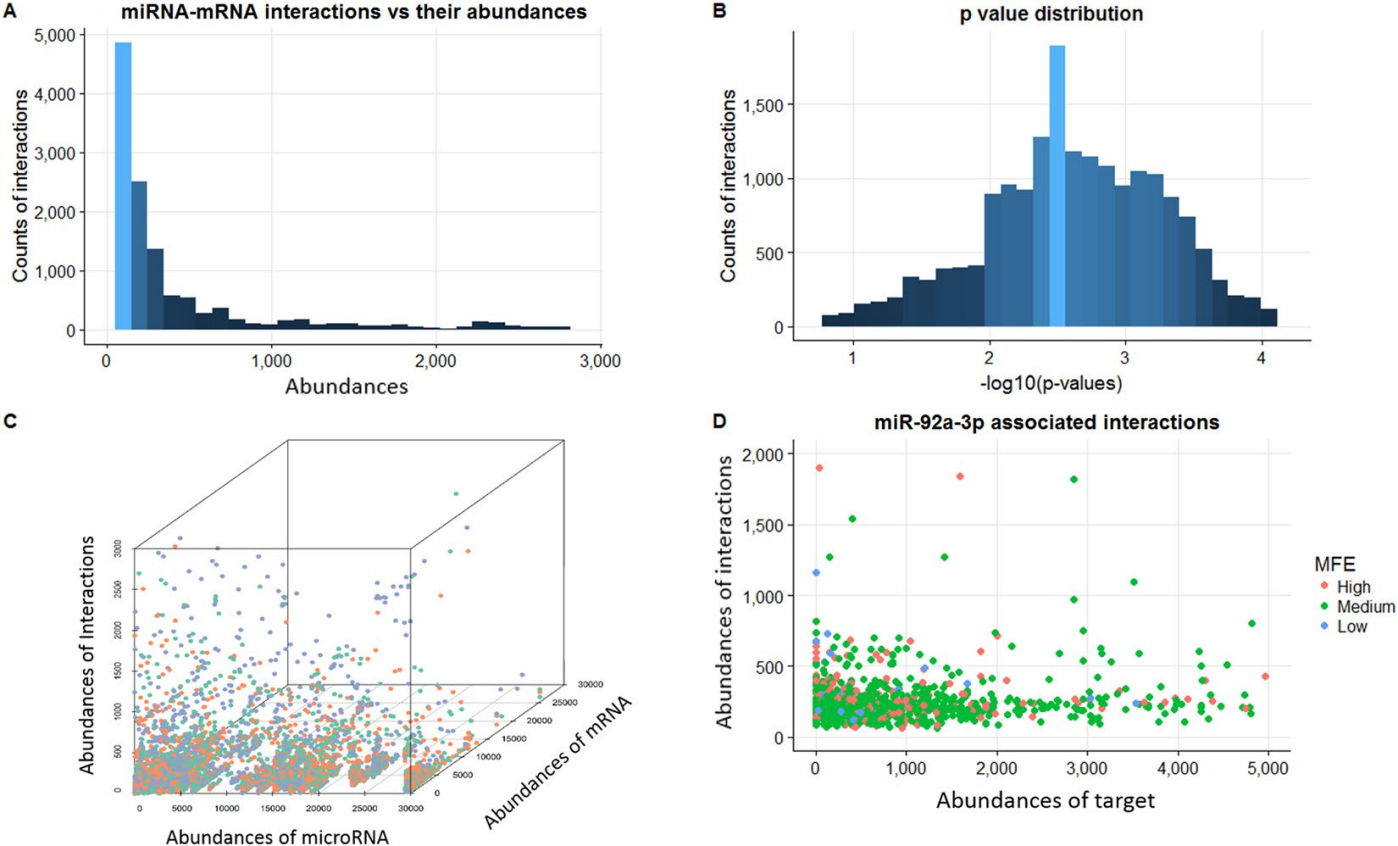
Overview of the interactions detected by the CLASH analysis. (**A**) Distribution of the miRNA-gene interactions (y-axis) with respect to their abundances (x-axis). (**B**) Distribution of the corrected p-values of each interaction from Binomial test for quality filtering. (**C**) Projection of each interaction based on the abundance of the corresponding miRNA (x-axis), mRNA (y-axis), and interaction (z-axis). (**D**) The relationship between the abundances of each miR-92a-3p interaction and the corresponding mRNA. The colored points indicated the three levels of binding affinities based on MFE values: High (red, [−31.2, −21.8]], Medium (green, [−21.8, 12.3]), and Low (blue, [−12.3, −2.9]).

After reanalyzing CLASH data (details in Methods), 17,400 miRNA-mRNA interactions resulting from 17,651 high-confidence binidng sites have passed the quality-filtering step and remained for the downstream analysis. Figure 2A shows the abundance distribution of these interactions and Fig. 2B shows the distribution of corresponding corrected *p*-values from the Binomial test. 95.3% of the interactions have their counts significantly higher compared to random interaction (*p*-value ≤ 0.05 after Bonferroni multiple test adjustment). Detailed statistics were provided in Supplementary Table S2.

We also observed weak correlations of miRNA regulatory interaction abundance with both mRNA and miRNA abundance (Fig. 2C). For example, 57.9% of miRNAs has Pearson correlation coefficient *r* ≤ 0.03 between the abundance of their target mRNAs and the number of interactions, contradicting the generally-believed assumption that miRNA binds to its different mRNA targets proportionally with respect to their sequence availability. Figure 2D shows that the most “versatile” miRNA, i.e., miR-92a-3p, regulates 956 different mRNAs through 1,065 different interaction sites. The interaction counts range from 63 to 23,870 (Mean = 296.5), significantly correlated with the corresponding mRNA abundance (*r* = 0.26). Similar weak correlations were also observed between binding affinities, e.g., minimum free energy (MFE)^51^, and interaction abundances (*r* = 0.01).

In summary, these findings suggest that in addition to mRNA sequence availability and binding affinity, there must be some other factors impacting the miRNA-mRNA binding, which makes miRNA regulation a highly selective process. For example, one possible factor can be mRNA structure that determines the accessibility for miRNA binding.

### MiRNA-mRNA interactions can be specific to cancer type and stage

The initial analysis of genomic data has identified differentially expressed (DE) genes and miRNAs in different stages of cancer (summary statistics are in Supplementary Table S3 while more details are available online). Many changes were found to be stage-specific. For example, there are 4,273 DE-genes with fold-change (FC) over 2 (fold) in at least one stage of KIRC, but only 21.2% (908) of them have more than 2-fold changes in all four stages. Similar observations were made on miRNAs, i.e., 174 DE-miRNAs were identified in KIRC and only 43.7% (76) were consistent across all stages. This analysis provides a list of 15,901 genes and 419 miRNAs that are cancer-associated for downstream analysis. However, this simple correlation analysis each time only checks one pair of miRNA and mRNA. It fails to acknowledge the fact that in reality there can be multiple miRNA regulators for one individual gene. Considering the fact of simultaneous multiple interactions, we turn to our proposed pipeline to solve this issue.

**Figure 3:**
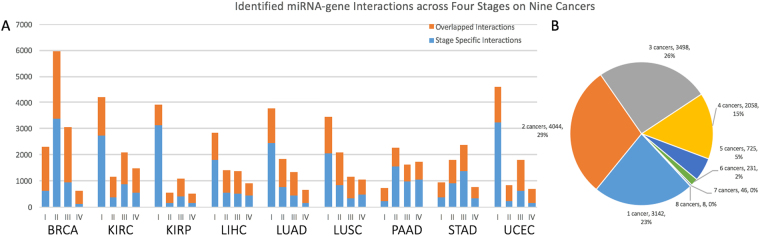
Overview of the miRNA-mRNA interactions identified in nine cancers. (**A**) Number of interactions detected in a specific stage (Blue) versus multiple stages (Orange), in each cancer. (**B**) Pie chart shows the percentages of interactions shared by different cancers.

**Figure 4:**
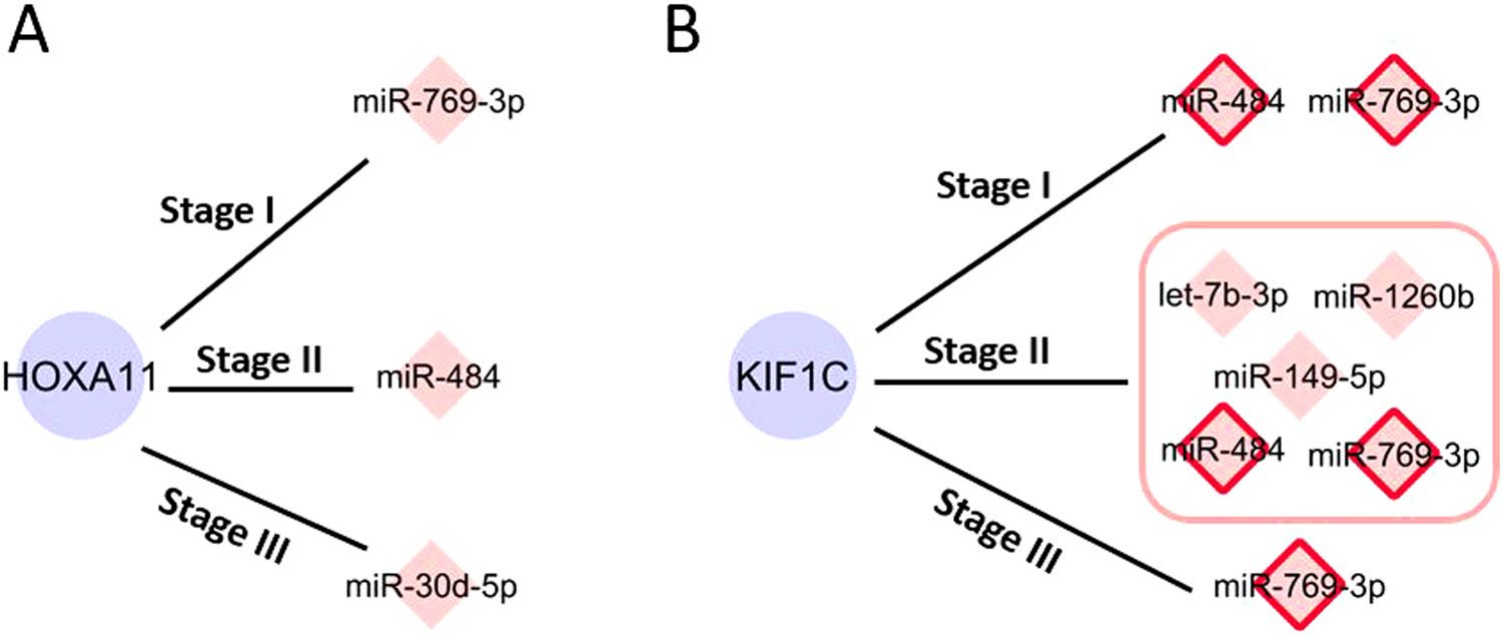
Illustration of the dynamic miRNA-mediated gene regulation (miRNA: pink diamonds and genes: purple nodes). (**A**) Three miRNAs regulate HOXA11 gene in different stages of KIRC. (**B**) Five miRNAs repress the same KIF1C gene in KIRC while the miR-484 and miR-769-3p (pink diamonds with red border) consistently interact with KIF1C across stages.

Using our meta-Lasso regression approach (Methods), we predicted interactions between miRNA and miRNA-targetable genes under each cancer developmental stage. Overall, there were 14,554 unique miRNA-mRNA interactions identified across all conditions. Similar to DE genes and miRNAs, those interactions tend to be cancer stage- or type- specific. Figure 3A shows the statistics of interactions identified in each cancer and each stage where the blue bars indicate the portion specific to the corresponding stage within that cancer type. First, we observed in most cancers (8 out 9), miRNA interactions are more abundant in early stages (Stage I + II) compared to the advanced stages, which highlights the miRNA’s implication in the early cancer progression. In total, 73.7% of the interactions are detected in only one stage of that cancer. In different stages of a cancer, the same miRNA may regulate different genes while the same gene can be regulated by different miRNAs. For example, a kidney developing gene, Homeobox A11 (HOXA11), was regulated by three miRNAs in KIRC and suppressed expression was observed during progression (3-fold, miR-769-3p (Stage I); 2.9-fold, miR-484 (Stage II); 2.1-fold, miR-30d-5p (Stage III); 2.2-fold, none (Stage IV)) (Fig. 4A). Similar observation was made on Kinesin Family Member 1 C (KIF1C) gene: five miRNAs interacted with KIF1C in three stages of KIRC (1.8-fold, miR-484 and miR-769-3p (Stage I); 1.9-fold, miR-1260b, let-7b-5p, miR-149-5p, miR-769-3p and miR-484 (Stage II); 2.0-fold, miR-769-3p (Stage III); 2.2-fold, none (Stage IV)) (Fig. 4B). This may indicate that different miRNAs can play a similar role in cancer development by targeting the same target.

In addition to the low consistency across stages of the same cancer, we also observed limited common miRNA regulatory interactions across cancers. Up to 92.7% (12,742) of the interactions were shared by less than five types of cancer and 22.8% (3,142) were identified specifically in one cancer type (Fig. 3B). Interestingly, even within the same tissue type, the number of common interactions across cancer subtypes was much lower than expected, e.g., 17.8% (1,264 out of 7,118) between KIRC and KIRP and 16.2% (1,051 out of 6,496) between LUAD and LUSC. This may be explained by the distinct expression profiles of genes and miRNAs in different subtypes of cancer. For example, KIRC and KIRP only share 34.5% of 6,332 DE genes and 23.5% of 136 DE miRNAs. The distinct miRNA regulatory patterns among stages and cancers demonstrate again the conditional feature of miRNA regulation. Moreover, it signifies that some miRNAs can only contribute to certain types or phases of tumor developments.

### Functional implication of miRNA regulation in kidney cancers

To assess the functional impacts of miRNA regulation in cancer progression, we further examined the interactions identified in two subtypes of kidney cancer (KIRC and KIRP), particularly focusing on those associated with cancer driver genes reported by Vogelstein *et al*.^52^. Per our identification, 19 cancer genes were regulated by miRNAs in both early-stage KIRC and KIRP (purple nodes in the upper panel, Figure S1), where only 16 out of the 118 related interactions are commonly shared by both cancers (red edges) and 12 genes are regulated by totally different miRNAs. For example, Neurogenic Locus Notch Homolog Protein 2 gene (NOTCH2) was regulated by miR-744-5p, -25-3p, -92a-3p, and -27b-3p in KIRC and by miR-744-5p, -106a-5p, -130b-5p, and let-7c-5p in KIRP. Another critical cancer suppressor gene TP53 was regulated by miR-454-3p in KIRC, and by miR-324-5p and -222-3p in KIRP. On the other hand, we observed that common miRNAs regulated different targets in two cancer subtypes. Among 27 common miRNAs, 65.0% of the interactions were linked to different cancer genes in both early-stage tumors.

Given the distinct mechanisms underlying the development of these two subtypes of cancer^53,54^, we are also interested in exploring how miRNA regulation contributes to the general or divergent growth of kidney cancers. Pathway and gene ontology (GO) enrichment analysis was performed using The Database for Annotation, Visualization and Integrated Discovery (DAVID v6.8)^55^ based on the suppressed genes in the early-stage cancers. Table 1 lists the top enriched KEGG pathways that are either common or specific in KIRC and KIRP (selected according to *p*-values and the complete list of GO and pathways are provided in Supplementary Table S4). Among the three common pathways (PI3K-ATK signaling pathway, Cell cycle, and Thyroid hormone signaling pathway), 22 genes were miRNA-regulated in the early-stage kidney cancer and 9 of them occurred in both subtypes. Only 7 miRNA-gene interactions were observed in both subtypes, which indicates that the same functional regulation can be realized by miRNAs interacting with different gene targets.

**Table 1:** Illustration of the common and cancer-specific pathways and the miRNA-mRNA interactions involved in KIRC and KIRP.

	Enriched Pathways	Interactions in KIRC	Interactions in KIRP
**Common**	PI3K-ATK Signaling Pathway	**MYC-miR-17-5p; TSC1-miR-320a; PPP2R1A**-miR-106a-5p/ -941; **TP53**-miR-454-3p; JAK1-miR-15b-5p; JAK2-miR-195-5p; PTEN-miR-181b-5p	**MYC-miR-17-5p**/ -320a/ -423-5p/ -744-5p/ let-7b-5p; **TSC1-miR-320a; PPP2R1A-miR-17-5p**/ -222-3p/ -92a-3p; **TP53**-miR-222-3p/ -324-5p; CCND1-miR-15a-5p/ -193b-3p/ -196a-5p/ -92a-3p; FGFR2-miR-186-5p; MAP2K1-miR-93-3p; NRAS-miR−877-3p; STK11-**miR-17-5p**
	Cell Cycle	**CREBBP-let-7e-5p/miR-1301-5p; MYC-miR-17-5p; TP53-miR-454-3p; EP300**-miR-30c-5p; ATM-miR-3144-3p/ -**92a-3p**; CDKN2A-miR-455-3p; STAG2-**miR-193b-3p**; ABL**1-let-7b-5p**	**CREBBP-let-7e-5p/ miR-1301-5p**/ -935; **EP300-let-7b-5p**/ -**193b-3p/ -92a-3p; MYC-miR-17-5p**/ -320a/ -423-5p/ **-744-5p/let-7b-5p; TP53**-miR-222-3p/ -324-5p; CDKN2A-miR-320a; CCND1-miR-15a-5p/ -**193b-3p**/ -196a-5p/ -92a-3p
	Thyroid Hormone Signaling Pathway	**CREBBP-let-7e-5p/miR-1301-5p; MYC-miR-17-5p; NCOA3-miR-20a-5p; NOTCH2-miR-744-5p/** -27b-3p/ -25-3p/ **-92a-3p; TP53-miR-454-3p; EP300**-miR-30c-5p; CTNNB1-miR-744-5p	**CREBBP-let-7e-5p/miR-1301-5p**/ -935; **MYC-miR-17-5p**/ -320a/ -423-5p/ **-744-5p**/ let-7b-5p; **NCOA3-miR-20a-5p; NOTCH2-miR-744-5p**/ -106a-5p/ -130b-5p/ let-7c-5p; **TP53**-miR-222-3p/ -324-5p; **EP300**-let-7b-5p/ -193b-3p/ -**92a-3p**; CCND1-miR-15a-5p/ -193b-3p/ -196a-5p/ -92a-3p; NRAS-miR-877-3p; MAP2K1-miR-93-3p; MED12-**miR-454-3p**
**KIRC-Specific**	Herpes Simplex Infection	**CREBBP-let-7e-5p**/miR-1301-5p; **TP53**-miR-454-3p; **EP300**-miR-30c-5p; JAK1-miR-15b-5p; JAK2-miR-195-5p; PTEN-miR−181b-5p; PTPN11-miR-130b-3p/ −183-5p/ −193b-3p	
	Transcriptional Misregulation	**MYC-miR-17-5p; TP53**-miR-454-3p; ATM-miR-3144-3p/ −92a-3p; CEBPA-miR-744-5p; H3F3A-miR-100-5p; MEN1-miR-17-5p	
	Jak-STAT Signaling Pathway	**CREBBP-let-7e-5p/ miR-1301-5p; MYC-miR-17-5p; EP300**-miR-30c-5p; JAK1-miR-15b-5p; JAK2-miR-195-5p; PTEN-miR-181b-5p; PTPN11-miR-130b-5p/ -183-5p/ -193b-3p	
**KIRP-Specific**	MAPK Signaling Pathway		**MYC-miR-17-5p/** -320a/ -423-5p/ -744-5p / let-7b-5p; **TP53**-miR-222-3p/ -324-5p; BRAF-miR-191-5p / -744-5p; FGFR2-miR-186-5p; MAP3K1-let-7g-5p/ miR-423-3p; MAP2K1-miR-93-3p; NRAS-miR-877-3p
	Rap1 Signaling Pathway		**GNAS**-miR-331-3p; BRAF-miR-191-5p/ -744-5p; FGFR2-miR-186-5p; MAP2K1-miR-93-3p; NRAS-miR-877-3p
	Central Carbon Metabolism		**MYC-miR-17-5p/** -320a/ -423-5p/ -744-5p/ let-7b-5p; **TP53**-miR-222-3p/ -324-5p; FGFR2-miR-186-5p; MAP2K1-miR-93-3p; NRAS-miR-877-3p

*Underline indicates the common interactions between KIRC and KIRP and **Bold** denotes the same genes or miRNAs involved in KIRC and KIRP.

**Figure 5:**
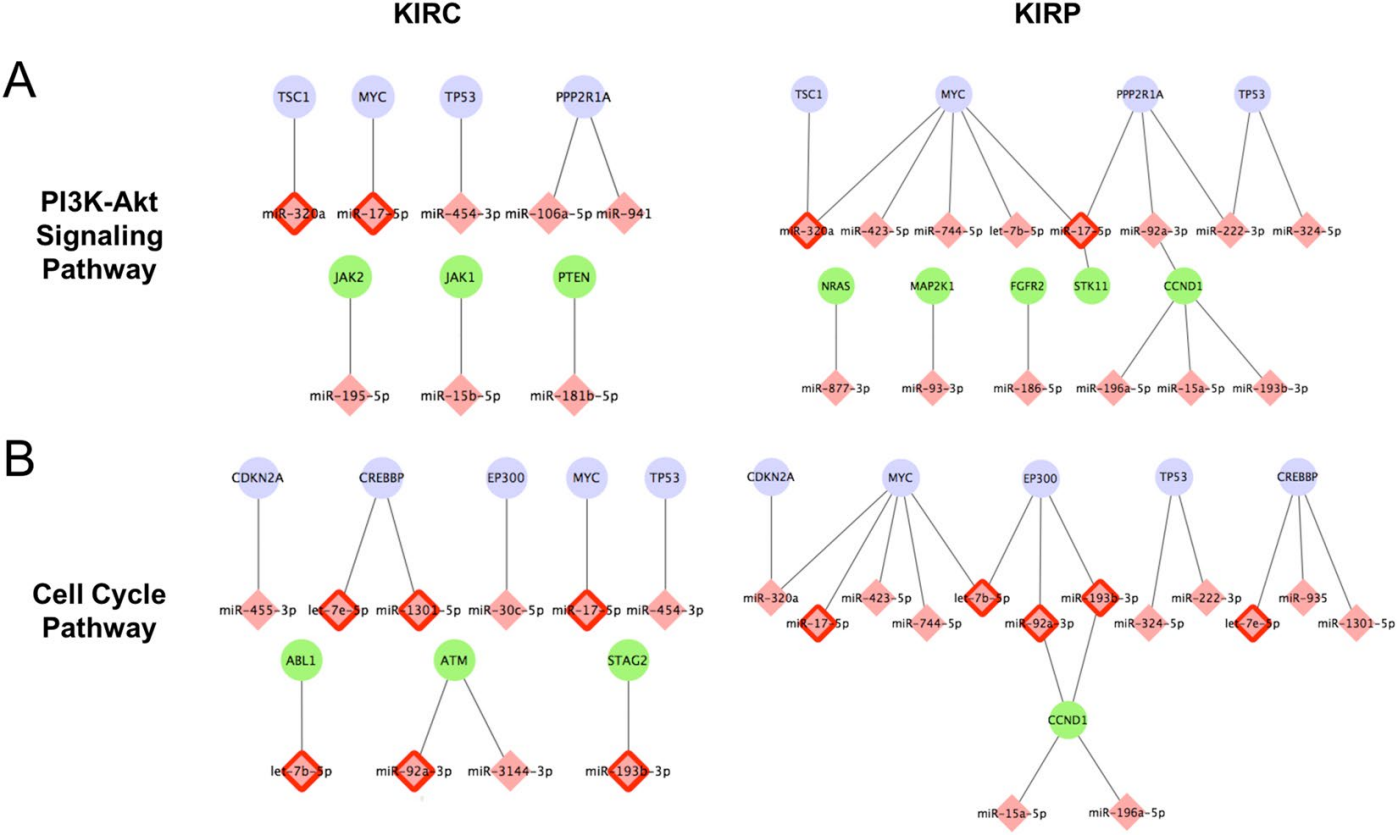
MiRNA-mediated gene regulation involved in two functional pathways in the early stage of KIRC and KIRP (miRNAs: pink diamonds; genes: ellipses). (**A**) PI3K-Akt Signaling Pathway; (**B**) Cell Cycle Pathway. Purple nodes denote the common genes between KIRC and KIRP; pink diamonds with red border indicate the common miRNA regulators; green ellipses are the condition-specific cancer genes.

Figure 5 demonstrates how miRNAs regulated PI3K-Akt signaling pathway (in 5 A) and cell cycle pathway (in 5B) in early-stage KIRC and KIRP through various conditional interactions. For example, the common genes (purple nodes), e.g., TSC1, MYC, and CDKN2A, were targeted by different miRNAs in two cancer subtypes while the common miRNA regulators (pink diamonds with red border), e.g., miR-320a, -17-5p, and 92a-3p, can interact with different targets, which leads to a different set of interactions that control the same functional pathway. Moreover, within the cancer-specific pathways, the distinct miRNA regulation patterns (Table 1) also provide new insights into cancer subtyping in the miRNA regulation perspective. In addition, similar observations were found by comparing LUAD and LUSC (Supplementary Table S5).

### Prediction of miRNA regulatory modules

Based on consistent observations in all nine cancers, we hypothesized that miRNAs can exert a regulatory role in certain biological functions through interacting gene targets that may be different across conditions. Moreover, multiple miRNAs can effectively cooperate in the same functional pathway by repressing the same or associated targets. Therefore we conducted a module detection based on the miRNA-gene interactions derived from the previous steps (Methods). As a result, 4,134 miRNA modules were identified and each included a set of 4–5 miRNAs consistently co-regulating the same set of pathways across more than two cancer conditions. Table 2 lists the summary statistics on the derived modules and details are provided in Supplementary Table S6.

**Table 2:** Statistics of the miRNA regulatory modules.

Module sizes (k)	# of k-miRNA Modules	# of modules reflecting significant pathways	# of consistent modules across >= 2 conditions				
			Total	Cancer-Specific		General	
				In 2 stages of a cancer	In > 2 stages of a cancer	In 2 cancers	In > 2 cancers
**1**	285	4	—	—	—	—	—
**2**	6,276	641	—	—	—	—	—
**3**	149,449	15,383	—	—	—	—	—
**4**	229,801	23,044	**2**,**349**	2,127	—	219	3
**5**	1,711,669	83,711	**1**,**785**	1,728	—	57	—

**Figure 6:**
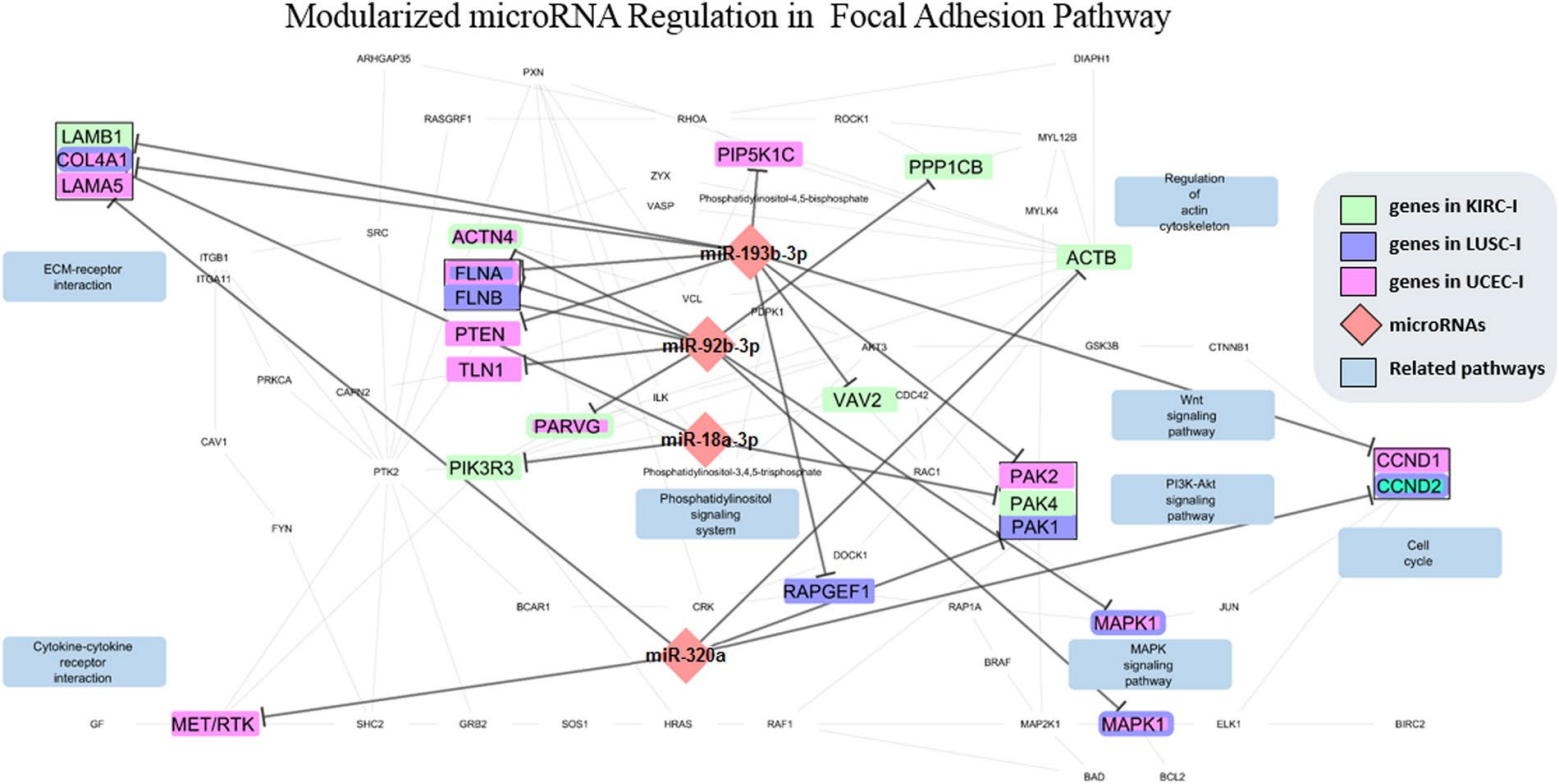
Illustration of the cooperative regulation of a miRNA module that contains four miRNAs: miR-18a-3p, -320a, -193b-3p, and -92b-3p (pink diamonds). This miRNA module consistently regulates the Focal Adhesion pathway in KIRC, LUSC, and UCEC. The target genes are color-coded (each color represents one cancer condition and the bi-colored nodes indicate the corresponding genes have been regulated by miRNAs in two conditions). The detailed miRNA-gene interactions are listed in Table 3. The network is visualized using Cytoscape 3.2.0 ^72^ with KEGGscape^73^ plugin.

We observed that most miRNA modules play their regulatory roles in different stages of cancer, and that only a small proportion is common across cancers. As expected, we observed in each single module that miRNAs coregulate one or multiple module, miRNAs co-regulate one or more biological processes through co-binding to the same target or different targets in the same pathway across conditions. Table 3 provides an example of a 4-miRNA module that regulates two pathways (glycolysis and focal adhesion) in three cancers (KIRC, LIHC, and UCEC). Under different conditions, miRNAs tend to target different genes but miR-320a always interacts with the same gene (PKM2) when regulating the glycolysis pathway. Figure 6 illustrates how the same miRNA module regulates critical signaling transduction genes in the focal adhesion pathway. For example, MET and LAMB1/COL4A1/LAMA5 were regulated in different stages. Since those genes are the initial activators and triggers in focal adhesion, the regulatory model is capable of controlling the whole process more efficiently by each miRNA member targeting different genes under different stages.

**Table 3:** An illustration on a 4-miRNA module (miR-18a-3p, -320a, -193b-3p and -92b-3p) that regulates two signaling pathways in three conditions. Gene targets are listed for each pathway and each condition.

Pathways	MicroRNAs	Conditions (Cancer/Stage)		
		KIRC	LIHC	UCEC
		Stage 1	Stage 2	Stage 1
**Glycolysis / Gluconeogenesis Pathway**	miR-18a-3p	PGK1, PKM2	PKM2	PKM2
	miR-320a	PKM2	PKM2	PKM2
	miR-193b-3p	GPI, PGAM1	GPI, ALDH3A2, ALDH7A1	GPI, ALDH3A2, TPI1
	miR-92b-3p	PGAM1	ALDOA	PGAM1
**Pathways**	**MicroRNAs**	**KIRC**	**LUSC**	**UCEC**
		**Stage 1**	**Stage 1**	**Stage 1**
**Focal Adhesion**	miR-18a-3p	PAK4, PIK3R3	COL4A1	COL4A1
	miR-320a	ACTB, CCND2	PAK1, CCND2	LAMA5, MET
	miR-193b-3p	LAMB1	FLNA, RAPGEF1, VAV2	CCND1, PTEN, PAK2, PIP5K1C, COL4A1
	miR-92b-3p	ACTN4, PARVG, PPP1CB	FLNB, MAPK1	ACTN4, FLNA, MAPK1, PARVG, TLN1

### Validation of identified regulatory interaction in cancers

Through comparison, 2,014 miRNA-gene interactions of our prediction have been annotated as experimentally validated entries in miRTarBase^56^, which are reported by various assays, such as Luciferase reporter assay, qRT-PCR or Western blot analysis with miRNA transfections (Supplementary Table S7).

To further validate the interations in cancer, we also performed an extensive literature search on all miRNA/cancer-related publications during 2001–2017 in PubMed database by utilizing the NCBI API tool. First, we searched for articles that mentioned the predicted miRNA- mRNA pairs in the corresponding cancer type, e.g., let-7a-5p-HMGA1 in breast cancer. Then we examined all 27,586 returned articles to confirm if the experimental evidence for a direct interaction is strong or not based on the experimental assays that have been used. Note that the negative expression correlation is not considered as strong evidence of direct interaction in our analysis. As a result, 43 miRNA-mRNA interactions associated with the nine types of cancer have been validated (Table 4). It is not surprising that those interactions are related to a few well-known cancer-associated miRNAs such as let-7a, miR-181a, and 200c, which reflects the reality that most of current experimental efforts are still on the assessment of well-reported targets in various cancer conditions. We believe computational prediction like ours can bring more new targets in this direction.

**Table 4:** Detailed information of validated interactions from literatures.

**microRNAs**	**Genes**	**Cancers**	**Techniques**
hsa-let-7a-5p	DICER1	BRCA^74,75^	1, 4
	HMGA1		2, 4
hsa-miR-10a-5p	EPHA4	LIHC^76^	2, 4
hsa-miR-17-5p	HBP1	BRCA^77,78^	1, 2, 4
	MYC		
	UBE2C	STAD^79^	
hsa-miR-181a-5p	DDX3X	UCEC^80^	2, 4
hsa-miR-193b-3p	CCND1	PAAD^81^	1, 2, 4
	YWHAZ		
	CCND1	STAD^82^	3, 4
hsa-miR-196a-5p	FOXO1	LUAD^83^	1, 4
	HOXA7	LUAD^84^	3, 4
	HOXB8	LUAD/LUSC^84^	
	HOXC8		
hsa-miR-196b-5p	HOXC8	BRCA^85^	1, 3, 4
		LUSC^85^	
hsa-miR-200c-3p	ZEB1	BRCA^86^	1, 2, 3, 4
		KIRC/KIRP^87^	
		LIHC^88^	
		LUAD/LUSC^89^	
		PAAD^90^	
		STAD^91^	
		UCEC^92^	
hsa-miR-210-5p	ISCU	LIHC^93^	1, 3, 4
		LUAD/LUSC^94^	
hsa-miR-221-3p	ATXN1	BRCA^95–97^	1, 3, 4
	BCL2L11		
hsa-miR-222-3p	PPP2R2A	LUSC^98,99^	3, 4
hsa-miR-23b-3p	PSAP	LUSC^100^	1, 2, 3, 4
hsa-miR-26a-5p	HSPA8	BRCA^101^	2, 4
hsa-miR-30c-5p	MTDH	LUAD/LUSC^102^	
	RAB18	LUAD^103^	
hsa-miR-30d-5p	HOXA11	UCEC^104^	2, 4
hsa-miR-320a	MYC	LIHC^105^	1, 2, 4
	VDAC1	UCEC^106^	
hsa-miR-424-5p	WEE1	KIRC^107^	1, 2, 4
hsa-miR-744-5p	TGFB1	KIRC/ KIRP^108^	2, 4

**Validation Techniques:** (1) Luciferase reporter assay, (2) quantitative RT-PCR, (3) Western blot analysis, (4) miRNA transfection.

### Model Quality Assessment

**Figure 7:**
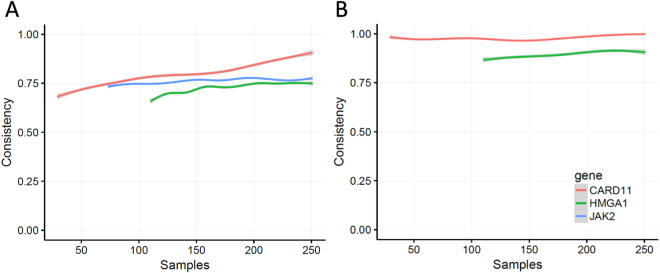
Consistency assessments of regulator selection regarding difference sample sizes of three genes: CARD11 (red, 28 potential regulators), JAK2 (blue, 73), and HMGA1 (green, 109). The lines denote the transition Jaccard’s similarity of the regulators selected at different sample sizes of these genes. (**A**) Consistency of all regulator selection across difference sample sizes. (**B**) Consistency of microRNA regulator selection across difference sample sizes.

Robustness of the proposed method was assessed based on the consistency of regulator detection across datasets with various sample sizes. Take the 251 samples from stage I of KIRC as an example. For each size i (5<=i<= 251), we randomly generated or each integer *i* from 5 to 251 (maximum sample size), we randomly generated 1000 testing data sets with the sample size. We conducted regulator selection for each gene based on the final model in the meta-Lasso regression analysis on each dataset. The consistency across models with different sample sizes was evaluated by comparing the similarity of the selected regulators. Figure 7A shows the comparisons on these selected regulators based on three genes (CARD11, JAK2, and HMGA1), and a high-level stability of our model with the increasing sample size was observed. Moreover, for miRNA regulator identification (Fig. 7B), we found that our method has reached the high-level consistency for CARD11 and HMGA1 (JAK2 were not regulated by miRNAs in this stage).

**Figure 8:**
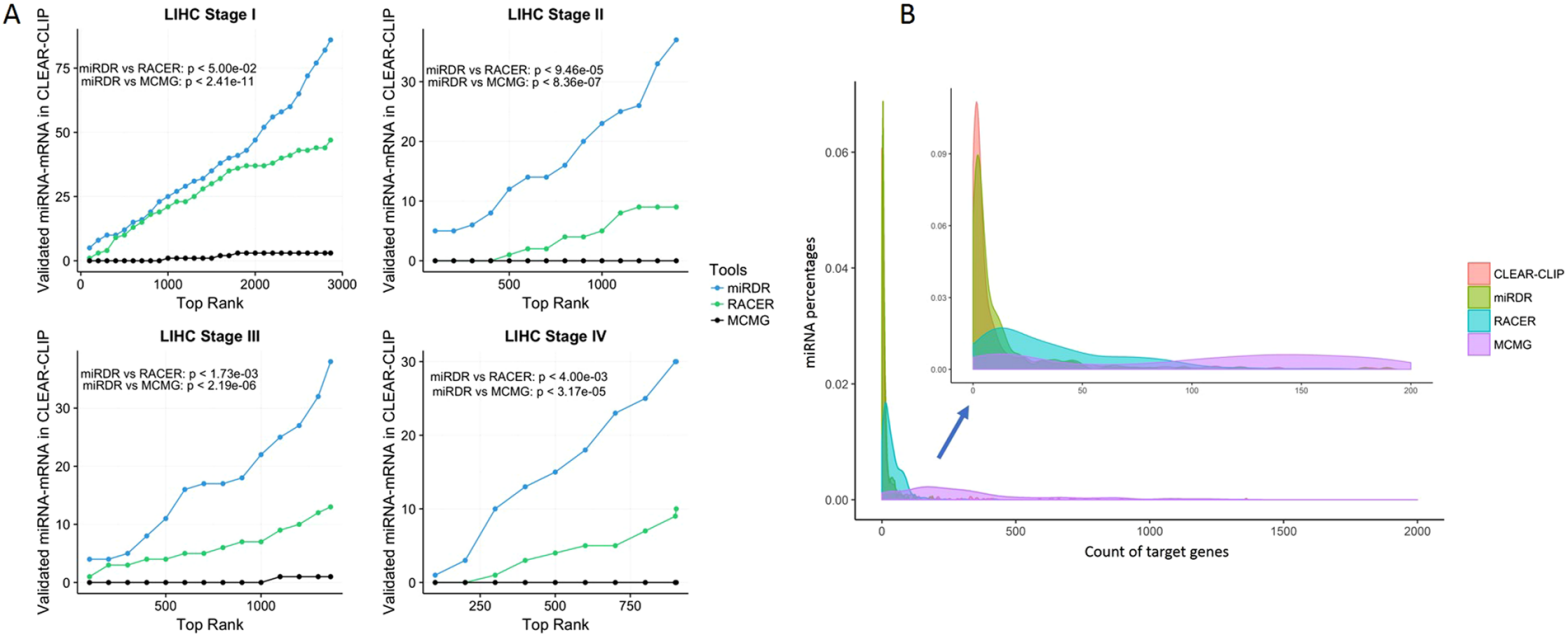
(**A**) Method comparison on the identified miRNA-target interactions on the test set. MiRDR, RACER and MCMG were applied on the Hepatocellular carcinoma (LIHC) data. The x-axis represents the top ranked interaction predicted by miRDR while the y-axis represents the count of validated interactions. The p-values were calculated by one-sided Wilcoxon singed rank test; (**B**) Method comparison based on the counts of identified miRNA targets. X-axis: number of target genes; y-axis: miRNA percentage. Outer plot shows the overall distribution and the inner shows the zoom-in view of partial distribution in the range of [0, 200].

To further demonstrate the performance of miRDR, we also conducted a peer comparison with two other well-established methods: RACER^57^ and MCMG^58^, on miRNA target prediction using expression data. The test dataset was collected from 332 patients in LIHC, available at the TCGA website^59^, along with 34,986 experimental miRNA-target interactions detected by CLEAR-CLIP in Hepatocellular carcinoma cell (Huh-7.5)^22^. We utilized three methods to predict the miRNA-target interactions in each stage of LIHC. First, we found that RACER and MCMG predicted significant more interactions than miRDR (average number of predicted interactions: miRDR 1,634; RACER 10,371; MCMG 212,171) but less experimental validated interactions among the top ranked ones. When taking a close look at each stage, we ranked the predicted interactions from RACER and MCMG according to the score and compare with top-n predictions from miRDR at 100-intervals (Fig. 8A). It is clear that miRDR outperformed two other methods by identifying significantly more validated interactions in each stage (p-value of one-sided Wilcoxon singed rank test is shown in the figure). Moreover, unlike RACER and MCMG, miRDR’s performance was not affected by the input sample size. For example, RACER and MCMG predicted only half validated targets in smaller sets (in stage II (82), III (80) and IV (6)) than what they did in a bigger set (stage I (164)) among top-1000 predictions. At another evaluation, we compared the distribution of miRNA target count predicted by three methods, using the experimental data from CLEAR-CLIP as reference. As Fig. 8B shows, the distribution of miRDR is very close to the experimental one, while RACER and MCMG tend to predict too many more targets for most miRNAs.

Although Lasso regression method is capable of handling the problem of more variable than samples^60^, we still recommend a minimum number of samples for each gene for using our meta-Lasso regression model in order to reach high confidence in the inferred miRNA regulators. Note these thresholding values also depend on the numbers of possible regulators each gene can have (Supplementary Table S8).

### Software and database access

We have developed a microRNA Dynamic Regulation Database (miRDR) that archives all the identified miRNA regulatory modules in each cancer online at http://sbbi.unl.edu/miRDR. An R package implementing the proposed method is available for download from our miRDR webpage.

## Discussion

In this study, we presented two important characteristics of miRNA regulation. ***Dynamic*** regulation refers to the fact that the same miRNAs interact with different targets under different conditions. Theoretically speaking, factors contributing to the dynamic property of miRNA regulation may include the distinct expression of miRNA and their target, potential SNPs involved in the binding site, DNA translocation in the gene region, competitive binding between miRNA and other RNA binding proteins. On the other hand, ***modularized*** regulation describes that miRNAs may cooperate when they target to the same gene or same functional process across different conditions. Intuitively speaking, when multiple miRNAs target the same process, especially when multiple genes are controlled (Fig. 6, Table 5 and Supplementary Table S6), they render higher efficacy in terms of functional regulation.

**Table 5:** Detailed statistics of samples from nine cancer types.

Cancer Types		Normal	Stage I	Stage II	Stage III	Stage IV
Breast invasive carcinoma	BRCA	104	181	601	242	20
Kidney renal clear cell carcinoma	KIRC	71^a^	251	55	125	80
Kidney renal papillary cell carcinoma	KIRP	71^a^	169	22	49	14
Liver hepatocellular carcinoma	LIHC	50	164	82	80	6
Lung adenocarcinoma	LUAD	58^b^	276	122	84	25
Lung squamous cell carcinoma	LUSC	58^b^	227	151	80	6
Pancreatic adenocarcinoma	PAAD	4	21	147	4	4
Stomach adenocarcinoma	STAD	32	55	119	174	42
Uterine Corpus Endometrial Carcinoma	UCEC	33	331	48	121	28

^a^The same set of normal samples were used in two type of Kidney cancers;

^b^The same set of normal samples were used in two type of Lung cancers.

Through heterogeneous genomic data integration, modeling, and adjustments of gene expression based on other regulatory mechanisms such as TF and CNV, we have detected 14,554 conditional miRNA-gene interactions in 9 types of cancer. The discrepancy between the inferred interactions from our pipeline and the CLASH-reported interactions may be explained by the fact that the former reported conditional interactions highly associated with cancers while the latter reported interactions associated with AGO1 protein in HEK293 cell. To generalize the CLASH reported miRNA-gene interactions to different cell types, we also designed a filtering process to minimize the possibility of overuse. Gene expression and miRNA expression have been assessed to ensure both molecules have a meaningful expression in the cell of interest. We also checked if any somatic mutation occurs at the CLASH reported binding site on mRNA sequence in the new cell line. The design of miRNA regulatory score enables an efficient differentiation among miRNA-mRNA interactions. In contrast to the existing methods that evaluate the interactions based on simple features such as binding affinity or complementary sequences, our miRNA scoring system learned and considered more features directly from experimental data, which is expected to be more reliable.

Comparing with two leading algorithms for miRNA target prediction based on expression profiles, miRDR has showed its advantageous predictive power with two major improvements. First, comparing with other methods, miRDR’s predictions tend to include more validated interactions while not introducing significant high false prediction. This is an important aspect given the widely-accepted critique on high false positive rate in current target prediction. Another advantage was the stable performance of miRDR on fairly limited sample size. The meta-analysis design minimized the impacts of sample variation on the target prediction, which seems promising to facilitate the detection of weak signals between miRNA and targets with insufficient samples.

Overall, our knowledge of modularized miRNA regulation is still very limited. Along this line, Ding *et al*. identified 181 modules based on a simple statistical analysis on CLASH reported interactions^24^. In this study, we have identified 4,134 functional miRNA modules based on the consistent miRNA co-regulation across different cancer conditions, where all previously reported modules are covered. Note the previous study focused on miRNAs that target the same gene while our method assesses comprehensively all modules involved in similar functional processes, which therefore can be more general. For example, one novel 5-miRNAs module, namely miR-196a-5p, -196b-5p, -365a-3p, -378a-5p and -30b-5p, has been identified to regulate cell cycle and MAPK/Wnt singling pathways in kidney and uterine cancers in our study. It was also conceived as a functional module by Trajkovski *et al*. where they uncovered that these miRNAs cooperatively promote the brown adipogenesis pathway in mice^61^.

In addition, since new sequencing technologies such as CLASH and CLIP can produce non-overlapped data on miRNA interactome, future inclusion of interactions detected in different platforms and cell types is expected. The concern of minimum sample size for reliable detection of regulators for each gene using our meta-lasso regression approach is actually a common concern of all similar analysis. We envision controlling the initial set of regulators including both TFs and miRNAs may alleviate this concern.

## Conclusion

In summary, we proposed an integrative computational method to identify reliable miRNA-gene interactions in a given biological condition based on the integration of heterogeneous genomic data. In order to stratifying complex gene regulation networks, our approach considers other gene-expression regulatory factors including TF regulation, CNV, and DNA methylation. Applying the meta-lasso regression approach to 9 types of cancer, we have examined in-depth all possible miRNA-mRNA interactions and regulatory modules that are involved in different phase of cancer progression. The observed properties of the identified interactions further demonstrated the dynamic feature of miRNA regulation during tumor progression. We hope the method developed in this study can provide the community a promising solution to overcoming scientific limitations in the study of dynamic miRNA regulation in complex systems by generating novel insights in dynamic targeting and dramatically reducing the extensive lab-load in related research areas.

## Methods

### Data preparation

Data used in this study are all publically available, including (1) raw sequencing reads of miRNA-mRNA interactions from the CLASH experiment^21^ (GSE50452); (2) DNA binding data of 150 TFs from large-scale ChIP-Seq analysis by the Encyclopedia of DNA Elements (ENCODE) Consortium^45^; (3) multi-level genomic data from 4,206 patients and 9 types of cancer from Cancer Genome Atlas (TCGA)^62^ including miRNA and mRNA expressions, CNVs, and DNA methylation data (Table 5). In addition, for each cancer patient, stage and subtype information was also retrieved from TCGA.

### CLASH data reanalysis

To assess the detection confidence level of CLASH reported binding sites, we first reanalyzed the raw sequencing data from CLASH and performed reads mapping against human reference genome (HG19) and human mature miRNA sequences database (miRBase^63^, version 21). Then, three types of reads were examined for each miRNA-mRNA binding site in the CLASH experiment including (1) chimeric reads that represent miRNA-mRNA binding sites; (2) miRNA single reads that represent free miRNAs, which are unbound with any mRNAs; (3) mRNA single reads that represent unbound mRNA sequences, which are unbound with any miRNAs.

Binomial test was applied on each binding site to infer the detection confidence level of a specific binding site *k* between miRNA *i* and the mRNA *j*. Specifically, the chimeric reads count of interactions between miRNA *i* and mRNA *j* at the binding site *k*, *C*_*ijk*_ was assumed to follow a binomial distribution ${C}_{ijk}\sim Binomial\,({C}_{all},\,{P}_{ijk})$ under the null hypothesis, where *P*_*ijk*_ (equation (2)) represents the probability of a binding occurred between miRNA *i* and mRNA *j* at the binding site *k*. Binomial test was performed and the *p*-value for each miRNA-mRNA binding site was calculated by the formula

1 $$p-valu{e}_{ijk}=\sum _{{C}_{ijk}}^{{C}_{all}}(\begin{array}{c}{C}_{all}\\ {C}_{ijk}\end{array}){P}_{ijk}^{C}(1-{P}_{ijk}{)}^{{C}_{all}-C}$$

where *C*_*all*_ is the count of chimeric reads supporting all interactions and the probability of a binding occurred between miRNA *i* and mRNA *j* at the binding site *k* is estimated by

2 $${P}_{ijk}=\frac{{C}_{ijk}}{Su{m}_{i}}\times \frac{{C}_{ijk}}{Su{m}_{j}}$$

where *Sum*_*i*_ and *Sum*_*j*_ represent the total reads counts (chimeric reads and single reads) associated with miRNA *i* and mRNA *j*, respectively. Binding sites with Bonferroni-multiple-test-adjusted *p* values less than 0.05 were considered to be significant and included in the further analysis.

### Regulatory score (*RS*) calculation of each miRNA-mRNA pair

To assess the likelihood of interactions between a potential target mRNA and a miRNA, we designed a probability-based regulatory score. As introduced above, we first estimated the probability of an interaction event between miRNA *i* and mRNA *j* at the binding site *k* by the equation (2), which we denoted as *P*_*ijk*_.

Then, raw *RS* of each miRNA-mRNA pair was calculated by the aggregation of the binding probability and the binding affinity, e.g., represented by the MFE^51^ of the corresponding interactions, as follows:

3 $$R{S}_{ij}=\frac{1}{K}\sum _{k=1}^{K}(|MF{E}_{k}|\times {P}_{ijk})$$

where *MFE*_*k*_ represents the minimum free energy of the binding site *k* and *K* is the total number of different binding sites between miRNA *i* and mRNA *j*.

To mimic the situation of competition among mRNAs on the same miRNA, raw *RS* was normalized by the total number of potential targets associated with miRNA *i*:

4 $$R{{S}_{ij}}^{^{\prime} }=R{S}_{ij}/R{S}_{i}.$$

where *RS*_*i*_. denotes the set of raw *RS* between all target *j* and miRNA *i*.

### Regulatory score (*RS*) calculation of each TF-gene pair

Similar to miRNAs, a TF may also have more than one binding site on a target gene. The *RS* for the TF *t* and gene *j* based on the binding site *k* was estimated based on the distance (*d*_*tjk*_) between the binding site *k* and the transcription start site (TSS)^47,64^, as follows:

5 $$R{S}_{tjk}=\exp (-(\frac{1}{2}+\frac{4{d}_{tjk}}{{10}^{5}}))$$

Because a smaller distance (*d*_*tjk*_) is associated with a higher regulatory potential of TF, *RS* for all binding sites of a TF and gene pair can be aggregated as follows^47^:

6 $$R{S}_{tj}=1-\prod _{k=1}^{K}(1-R{S}_{tjk})$$

To mimic the situation that the same TF can bind to more than one gene, raw *RS* was normalized by the formula $R{{S}_{tj}}^{^{\prime} }=R{S}_{tj}/R{S}_{t}.$ where $R{S}_{t}.$ is the sum of $R{S}_{tj}$ for a fixed *t* over all *j*’s.

### Identification of the Conditional Gene Regulators through the Lasso Regression

**Figure 9:**
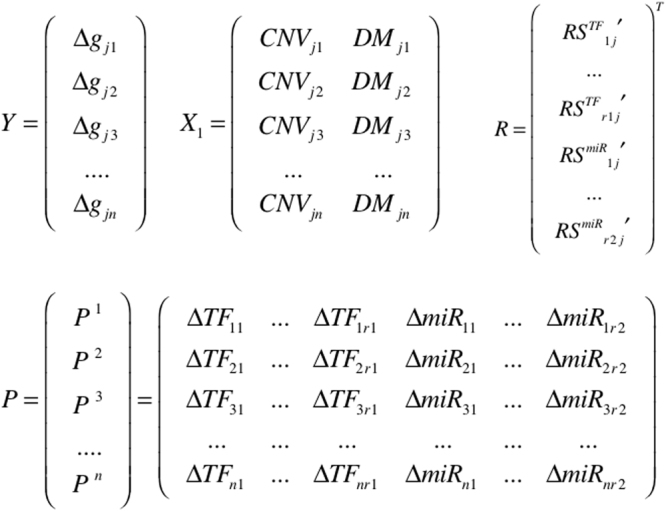
Four matrices constructed for each gene *j* and each condition, i.e., a specific cancer stage. *Y* represents the vector of expression changes of gene *j* in the corresponding cancer patients under this condition; *X*_1_ represents the matrix of background adjustment factors (CNV and DNA methylation); *R* represents a *RS* vector that contains the regulatory scores of all* r*_1_ TF- and *r*_2_ miRNA- regulators of gene *j*; *P* is the matrix that includes all the expression changes of each regulator in each patient under the corresponding condition.

In this study, we considered each stage of the nine types of cancer as an individual condition *D*_*sc*_ (stage: *s* = 1,2,3,4; cancers: *c* = 1,2,…,9). For each gene under a specific condition, four matrices were constructed for a lasso regression approach to identify the conditional TF and miRNA regulators (Fig. 9) including the following:

- The expression vector *Y* of size (*n* × 1) represents the expression changes of gene *j* in each of the *n* cancer patients associated with this condition versus healthy control. Here, in sample *n*, the expression changes of gene *j* and miRNA *i* were calculated by comparing with the average expression in the normal control group as follows:
  7 $${\rm{\Delta }}{g}_{jn}={\mathrm{log}}_{2}({g}_{jn}/\,\overline{{g}_{jcontrol}})$$
  8 $${\rm{\Delta }}{m}_{in}={\mathrm{log}}_{2}({m}_{in}/\,\overline{{m}_{icontrol}})$$
  - where $\,\overline{{g}_{jcontrol}}$ and $\,\overline{{m}_{icontrol}}$ denote the average expression levels of *g*_*j*_ and *m*_*i*_ among the normal control samples.
- The background matrix *X*_1_ of size *n* × 2 includes values of the copy number change and methylation of the gene *j* in each cancer compared to the healthy control;
- The regulator matrix *P* = [*P* ^1^,…, *P* ^*n*^]^*T*^ of size *n* × (*r*_1_ + *r*_2_) lists the expression changes of all regulators, including *r*_1_ TFs and *r*_2_ miRNAs, that interact with gene *j*, in each patient compared to the normal control;
- The regulatory score matrix *R* of size 1 × (*r*_1_ + *r*_2_) lists the *RS* of each corresponding regulator to the gene *j* derived from equations (4) and (6).

Based on the four matrices, a linear system is created as equation (9), where *X*_2_ = [*P* ^1^ × *R*, …, *P* ^*n*^ × *R*]^*T*^ (inner products of *R* and each row in *P*) represents the regulatory effects by TFs and miRNAs. The regulatory scores in *X*_2_ denote the impacts of corresponding regulator (column) to the gene in a specific sample (row). Before we solve the linear system in equation (9), the Frisch–Waugh–Lovell (FWL) method^47,65,66^ was applied to adjust for the gene expression change caused by the background factors (CNV and methylation) through a linear transformation, and convert the linear system into equation (10). Then, Lasso regression^60,67^ was used to identify the TFs and miRNAs that regulated the gene under the given condition.

9 $$Y={\beta }_{1}{X}_{1}+{\beta }_{2}{X}_{2}+\varepsilon $$

10 $${M}_{{X}_{1}}Y={M}_{{X}_{1}}{\beta }_{2}{X}_{2}+\varepsilon ^{\prime} ,\,where\,{M}_{{X}_{1}}=I-{X}_{1}{({{X}_{1}}^{T}{X}_{1})}^{-1}{{X}_{1}}^{T}$$

### Meta-Lasso regression for robust regulator identification

To minimize the bias caused by limited cancer samples from TCGA and enhance the robustness in regulator selection, meta-Lasso regression^68^ was adopted on regulator identification of each gene under each condition. The workflow is shown in Fig. 1B where a hierarchical sampling and model integration was performed. Li *et al*. demonstrated that the meta-Lasso method has the good consistency of selection and low false predication rate on several high-dimensional gene expression data^68^. Here, we adopted the objective function of their meta-Lasso regressions to detect the most likely regulators of each gene with the consideration of consistency across of multiple “individual” dataset:

11 $$\mathop{\max }\limits_{{\beta }_{1},g,\zeta }\{\sum _{m=1}^{M}{l}_{m}({\beta }_{{m}_{0}},g,{\zeta }_{m})-w\sum _{p=1}^{P}|{g}_{p}|-\lambda \sum _{p=1}^{P}\sum _{m=1}^{M}|{\zeta }_{mp}|\}$$

where ${l}_{m}({\beta }_{{m}_{0}},g,{\zeta }_{m})$ is the log-likelihood function of the *m*-th dataset; *M* denotes the number of individual datasets; *g*_*p*_ is the effect of the *p*-th regulator (out of *P* regulators) at the overall condition; and *ζ*_*mp*_ is the effect of the *p*-th regulator at the *m*-th dataset (out of *M* datasets).

Specifically, among the samples under one cancer condition, two rounds of 5-fold data separation were performed to generate 25 individual subsets that each contains a randomly selected 64% (80% × 80%) of the entire samples (Fig. 1B). The final LASSO regression model was achieved by applying the meta-LASSO method to the hierarchical sampled subsets. Any none-zero regression coefficients in the final LASSO model indicate the interactions occurred between the corresponding regulators and genes.

Subsequently, the same analysis was conducted for every human gene *j* in 36 conditions across all cancers (4 × 9). As shown in the workflow (Fig. 1), we followed a hierarchical framework to identify cancer-specific regulations (based on the consistency across different stages of that cancer) and common cancer regulation (based on the consistency across all cancers) through similar model selection procedure as described above.

### Assessment of the model stability through a robustness test

To assess the robustness of this model, particularly its dependence on the sample size, we performed regulator identification with different sample sizes from p (the total number of potential regulators) to the total number of patients under that condition. We randomly generated 1,000 sets of data to perform the analysis at each sample size *n*, then evaluate the consistency of the derived models by calculating the Jaccard’s similarity coefficient between selected regulators (*L*) between model *F*_*n*_ (regulator selection using *n* samples) and its neighbor *F*_*n-1*_ (using *n*-1 samples), as follows:

12 $$J({F}_{n},\,{F}_{n-1})=\frac{|L({F}_{n})\,\cap L({F}_{n-1})|}{|L({F}_{n})\,\cup L({F}_{n-1})|}$$

### Assessment of potential miRNA-gene interactions in a specific cancer

Although the reported miRNA-gene interactions, e.g., 37,751 from miRTarBase^56^ database were validated in a particular experiment on a certain type of cell, the same physical binding between the participating miRNA and gene can still be valid in other cells as long as their sequences and expressions are conserved. With appropriate processing, we believe the interactions obtained from one experiment can be conditionally generalized to other cell types. Specifically, for each interaction, we conducted the following checkup when applying it to a new cell type:

- both miRNA and gene must have meaningful expressions in the cell type of interest to maintain the activity level of the interaction;
- the annotated binding sites shouldn’t show any mutations in the new cancer cell. Otherwise, we would no longer consider this binding site in the regulatory score calculation step.

In this proof-of-concept study, we focused on the experimental-validated binding sites from CLASH due to the availability of raw sequencing read information that is required in our model for regulatory score calculation.

### Detections of miRNA regulatory module based on functional involvement in cancers

Based on the conditional miRNA-gene interactions derived from all cancers and stages, we explored the modularized miRNA regulation by examining their involvement in known functional pathways, i.e., 171 fundamental pathways from the KEGG database^69^. We exhaustively searched for all *k*-miRNA (*k* = 1–5) candidate modules where miRNAs consistently regulated the same set of pathways across multiple conditions and evaluated the significance in the following steps:

i. Under each condition, the miRNAs were linked to a set of KEGG pathways through their target genes. Then, the miRNAs were grouped by the commonality on the same pathway as candidate modules (*k* = 1–5);
ii. For each candidate module under one condition, a pathway enrichment analysis was conducted to ensure the involvement of each pathway is statistically significant through a test based on the hypergeometric distribution^70^:
  13 $$\Pr ({r}_{w}|{N}_{D},{p}_{w},n)=\frac{(\begin{array}{c}{p}_{w}{N}_{D}\\ {r}_{w}\end{array})(\begin{array}{c}(1-{p}_{w}){N}_{D}\\ n-{r}_{w}\end{array})}{(\begin{array}{c}{N}_{D}\\ n\end{array})}$$
  where *N*_*D*_ denotes the total number of miRNA-regulated genes in the condition *D*, *P*_*w*_ represents the percentage of human genes associated with pathway *w*, *n* indicates the total number of target genes associated with the module and *r*_*w*_ is the number of genes belong to pathway *w*.
iii. A Binomial test was applied to assess the statistical significance of the involvement of |*w*|-pathways in a miRNA module *M*
  14 $${w}_{MD}\sim Binomial(|W|,\,{P}_{WD})$$
  15 $${P}_{WD}=\frac{\sum _{w=1}^{171}{(TotalnumberofModuleswithsizek)}_{wD}}{|W|(\begin{array}{c}K\\ k\end{array})}$$
  where *k* denotes the number of miRNAs in the module while *K* is the total number of miRNAs in this analysis, i.e., 371; *P*_*WD*_ denotes the overall probability of a *k*-miRNA group co-involves a common pathway under condition *D*; *w*_*MD*_ represents the number of enriched pathways associated with the *k*-miRNA module *M* under condition *D*; | *W* | is the total number of functional pathways.
iv. Another Binomial test was utilized to examine the statistical significance of the recurrence of the candidate module *M* across conditions.

16 $${d}_{M}\sim Binomial(|D|,\,{P}_{k})$$

17 $${P}_{k}=\frac{\sum _{D=1}^{36}{(TotalnumberofModuleswithsizek)}_{D}}{|D|(\begin{array}{c}K\\ k\end{array})}$$

where *k* and *K* are defined in (14); *P*_*k*_ denotes the overall probability of a *k*-miRNA module associated with common pathways across conditions; *d*_*M*_ represents the number of conditions where the *k-*miRNA module *M* is associated with common pathways; |*D*| is the total number of conditions.

Finally, we calculated False Discovery Rate (FDR) *q*-values from *p*-values using Benjamini’s method^71^. Module candidates with *q*-values < 0.05 were reported as miRNA regulatory modules, i.e. we controlled FDR at 0.05 in our integrative report of all miRNA module candidates with different *k*’s.

## Electronic supplementary material

miRDR code

Supplementary Information

Supplementary Table S2

Supplementary Table S4

Supplementary Table S5

Supplementary Table S6

Supplementary Table S8

Supplementary Table S9
