# Supplementary material for: Dynamic and Modularized MicroRNA Regulation and Its Implication in Human Cancers
Source: Sci Rep. 2017 Oct 17;7:13356. doi: 10.1038/s41598-017-13470-5 (PMC5645395; doi:10.1038/s41598-017-13470-5)
Supplement: Supplementary file 2 — Supplementary Information [file 41598_2017_13470_MOESM2_ESM.pdf]

Dynamic and Modularized MicroRNA Regulation and Its Implication in Human Cancers

Jiang Shu[1], Bruno Vieira Resende e Silva[1], Tian Gao[1], Zheng Xu[2,3], Juan Cui[1,*]

[1]Systems Biology and Biomedical Informatics (SBBI) Laboratory, Department of Computer Science and Engineering, [2]Department of Statistics, [3]Quantitative Life Sciences Initiative, University of Nebraska-Lincoln, Lincoln, NE, 68588, USA

* Correspondence should be addressed to J.C. (email: jcui@unl.edu).

Supplementary Methods

Collection and pre-processing of Cancer Data from TCGA

The level-3 data on genomics information, including expression of mRNAs/miRNAs, copy number variation (CNV) and DNA methylation, were downloaded from the TCGA Data Portal[1] (July 2015). Data were processed as follows.

mRNA expression. The mRNA expression profile of each individual was obtained from TCGA Level-3 data (files with postfix *.genes.normalized_results and *gene.quantification* files). Expression profiles were merged as a matrix where rows represent samples and columns represent different mRNAs. For each mRNA expression matrix associated with a caner type, RSEM[2] normalization was applied before further analysis.

miRNA expression. Similar to mRNA expression, miRNA expression was also retrieved from TCGA Level-3 data (files with postfix *.isoform.quantification* files). The expression of mature miRNA was obtained using BCGSC miRNA profiling pipeline[3] based on the annotation from miRbase[4] (Version 21). For each cancer type, the miRNA expression matrix was built by merging all individual profiles and normalized using RSEM[2] normalization.

Copy Number Variation. The normalized copy number of genes for each sample was obtained from TCGA data files with postfix *SNP_N_GenomeWideSNP_*.[hg19|grch38].seg.txt. The corresponding human RefSeq Gene coordinate was retrieved from UCSC Genome Browser[5]. Each CNV event were converted to the matched RefSeq gene symbols using Bedtools[6]. Multiple CNV events within the same gene were averaged.

DNA Methylation. The beta values of each gene in each sample were downloaded from DNA methylation level-3 data (*HumanMethylation450.6.lvl-3* files). Multiple beta values of the same gene were averaged.

In addition, the clinical information (xml files) of each participant was also obtained from the TCGA Data Portal[1]. All samples were categorized according to the cancer/normal class and stage information. Sub-stages (e.g., IA, -IB, -IC) were grouped into main-stage level (e.g., stage-I). The complete list of TCGA participant ID was provided in Supplementary Table S9, which can be used for retrieving the entire dataset used in our analysis.

Supplementary Reference

1. Chang, K. *et al.* The Cancer Genome Atlas Pan-Cancer analysis project. *Nat Genet* **45,** 1113–1120 (2013).
2. Li, B. & Dewey, C. N. RSEM: accurate transcript quantification from RNA-Seq data with or without a reference genome. *BMC Bioinformatics* **12,** 323 (2011).
3. Chu, A. *et al.* Large-scale profiling of microRNAs for The Cancer Genome Atlas. *Nucleic Acids Res* (2015). doi:10.1093/nar/gkv808
4. Kozomara, A. & Griffiths-Jones, S. miRBase: annotating high confidence microRNAs using deep sequencing data. *Nucleic Acids Res* **42,** D68-73 (2014).
5. Rosenbloom, K. R. *et al.* The UCSC Genome Browser database: 2015 update. *Nucleic Acids Res.* **43,** D670–D681 (2015).
6. Quinlan, A. R. & Hall, I. M. BEDTools: A flexible suite of utilities for comparing genomic features. *Bioinformatics* **26,** 841–842 (2010).
7. Shannon, P. *et al.* Cytoscape: A software Environment for integrated models of biomolecular interaction networks. *Genome Res.* **13,** 2498–2504 (2003).
8. Lu, Y., Zhou, Y., Qu, W., Deng, M. & Zhang, C. A Lasso regression model for the construction of microRNA-target regulatory networks. *Bioinformatics* **27,** 2406–2413 (2011).

9. Muniategui, A. *et al.* Quantification of miRNA-mRNA Interactions. *PLoS One* **7,** e30766 (2012).
10. Chen, X., Slack, F. J. & Zhao, H. Joint analysis of expression profiles from multiple cancers improves the identification of microRNA-gene interactions. *Bioinformatics* **29,** 2137–2145 (2013).
11. Jacobsen, A. *et al.* Analysis of microRNA-target interactions across diverse cancer types. *Nat. Struct. Mol. Biol.* **20,** 1325–1332 (2013).
12. Balwierz, P. J. *et al.* ISMARA: automated modeling of genomic signals as a democracy of regulatory motifs. *Genome Res.* **24,** 869–84 (2014).
13. Li, Y., Liang, M., Zhang, Z., Luo, J. & Zhang, Z. Regression analysis of combined gene expression regulation in acute myeloid leukemia. *PLoS Comput Biol* **10,** e1003908 (2014).
14. Kazan, H. & Hilal. Modeling Gene Regulation in Liver Hepatocellular Carcinoma with Random Forests. *Biomed Res. Int.* **2016,** 1–6 (2016).

Supplementary Figure

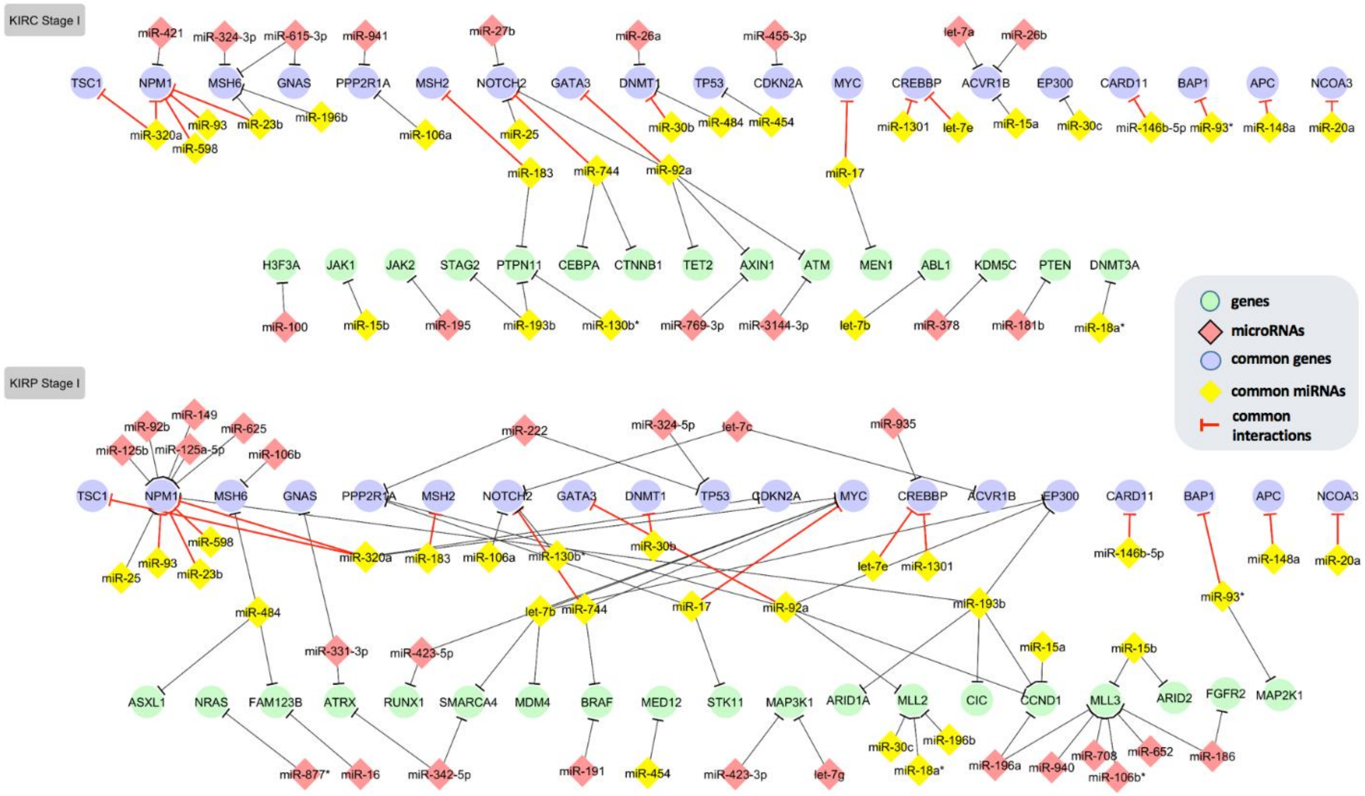

Supplementary Figure S1. miRNA-regulated cancer genes in the early stages of KIRC and KIRP. Green nodes represent miRNA-repressed cancer driver genes; purple nodes denote the cancer driver genes were shared by cancers; red diamonds represent the miRNAs while the yellow diamonds are the common miRNAs that regulate cancer genes under both conditions; red edges indicate that the corresponding miRNA-mediated gene interaction is shared by both. The network was generated in Cytoscape 3.2.0[7].

Supplementary Tables

Supplementary Table S1. Comparison of the computational models on miRNA regulation identification

Supplementary Table S2. The statistical significance of miRNA-mRNA binding sites at the filtering step

Supplementary Table S3. Statistics on the DE- genes and miRNAs in each stage of the nine cancers (with more than 2 FC expression change)

Supplementary Table S4. The miRNA-regulated pathways and gene ontology terms identified in the early-stage KIRC and KIRP

Supplementary Table S5. The miRNA-regulated pathways and gene ontology terms identified in the early-stage LUAD and LUSC

Supplementary Table S6. The microRNA regulatory modules detected in nine types of cancer, along with the functional pathways they regulated

Supplementary Table S7. Detailed information of validated interactions from literatures

Supplementary Table S8. List of regulators, including both TFs and microRNAs, identified for each gene

Supplementary Table S9. TCGA participant IDs of each cancer type used in the analysis

Table S1. Comparison of the computational models on miRNA regulation

Study	Integrated Regulatory Factors Other Than miRNA			miRNA-mRNA Interaction			Identification Model	Software Availability
	CNV	DM	TF					
Lu et al. 2011[8]	-	-	-	Predicted using TargetScan and PicTar	Cell specific expression	Static	LASSO Regression	Software download discontinued
Muniategui et al. 2012[9]	-	-	-	Mixed predicted and validated targets from multiple databases	Cell specific expression	Static	LASSO Regression	Software download discontinued
Chen et al. 2013[10]	-	-	-	Negative Correlation-based	Cell specific expression	Static	Empirical Bayes method	Standalone package: **MCMG**
Jacobsen et al. 2013[11]	✓	✓	-	Predicted using miRanda and TargetScan	Cell specific expression	Static	Multivariate linear regression model	No software available
Balwierz et al. 2014[12]	-	-	✓	Predicted using TargetScan	Non-specific	Static	Simple linear model	Webserver discontinued
Li et al. 2014[13]	✓	✓	✓	Predicted using TargetScan with number of binding sites	Cell specific expression	Static	Two-stage regression model	Standalone package: **RACER**
Kazan 2016[14]	✓	✓	✓	Predicted using TargetScan	Cell specific expression	Static	Random forests	No software available
**Shu *et al. 2017***	✓	✓	✓	Experimentally validated (e.g. based on CLASH)	Cell specific expression and binding sites	Static with integration of competitive regulation	Meta-Lasso regression models with Frisch-Waugh-Lovell method	Standalone package: **miRDR**, along with an online database

Table S3. Statistics on the DE- genes and miRNAs in each stage of the nine cancers (with more than 2 FC expression change)

Cancer Types	Stage I			Stage II			Stage III			Stage IV			Consistent across all stages		
	# of Genes	# of miRNAs	# of Interactions	# of Genes	# of miRNAs	# of Interactions	# of Genes	# of miRNAs	# of Interactions	# of Genes	# of miRNAs	# of Interactions	# of Genes	# of miRNAs	# of Interactions
**BRCA**	4,711	120	4,160	5,157	155	5,637	4,972	146	4,515	5,570	147	3,624	895	103	286
**KIRC**	4,975	111	4,754	5,294	125	7,772	5,820	110	4,262	6,015	130	7,212	908	76	411
**KIRP**	5,722	121	5,133	5,850	149	3,843	5,953	151	6,702	6,295	124	2,781	802	65	175
**LIHC**	4,242	150	4,342	5,356	147	7,320	5,810	190	7,414	5,089	163	1,352	670	89	133
**LUAD**	5,138	155	4,932	5,276	145	4,147	5,546	146	6,985	5,392	188	4,063	1,234	98	224
**LUSC**	6,948	211	4,765	7,029	206	4,035	7,237	227	7,440	6,923	145	1,501	881	100	115
**PAAD**	4,235	150	3,858	3,319	92	4,377	3,203	96	836	3,879	124	899	911	59	47
**STAD**	3,294	190	7,425	2,923	127	3,963	3,111	148	4,320	3,050	147	5,988	1,720	84	329
**UCEC**	6,244	255	5,242	6,098	267	7,210	6,222	287	4,123	6,638	281	5,322	910	203	356

Table S7. Detailed information of validated interactions from literatures

microRNAs	mRNAs	Cancers	PubMed IDs	Techniques	Evidences
hsa-let-7a-5p	DICER1	BRCA	26460550	1, 4	Figure 5
	HMGA1		25846193	2, 3, 4	Figure 7
hsa-miR-10a-5p	EPHA4	LIHC	22996586	2, 4	Figure 2
hsa-miR-17-5p	HBP1	BRCA	20505989	1, 2, 3, 4	Figure 3
	MYC	BRCA	18695042		Figure 6
	UBE2C	STAD	25760688		Figure 2
hsa-miR-181a-5p	DDX3X	UCEC	22492871	2, 4	Figure 5
hsa-miR-193b-3p	CCND1	PAAD	22188669	1, 2, 4	Figure 6
	YWHAZ				
	CCND1	STAD	27071318	3, 4	Figure 4
hsa-miR-196a-5p	FOXO1	LUAD	27880728	1, 4	Figure 7
	HOXA7	LUAD	19418581	3, 4	Figure 4
	HOXB8	LUAD/LUSC			
	HOXC8	LUAD/LUSC			
hsa-miR-196b-5p	HOXC8	BRCA	20736365	1, 3, 4	Figure 3
		LUSC			Figure 4
hsa-miR-200c-3p	ZEB1	BRCA	24615544	1, 2, 3, 4	Figure 4
		KIRC/KIRP	23754305		Figure 4
		LIHC	22735571		Figure 5
		LUAD/LUSC	17804704		Figure 3
		PAAD	26081037		Figure 4
		STAD	25502084		Figure 3
		UCEC	22685266		Figure 2
hsa-miR-210-5p	ISCU	LIHC	27983913	1, 3, 4	Figure 5
		LUAD/LUSC	25825391		Figure 1
hsa-miR-221-3p	ATXN1	BRCA	25686829	1, 3, 4	Figure 6
	BCL2L11		28191469, 26503209		Figure 4, Figure 3
hsa-miR-222-3p	PPP2R2A	LUSC	27602961, 21656127	3, 4	Figure 6, Figure 3
hsa-miR-23b-3p	PSAP	LUSC	24966325	1, 2, 3, 4	Figure 3
hsa-miR-26a-5p	HSPA8	BRCA	24735615	2, 4	Figure 3
hsa-miR-30c-5p	MTDH	LUAD/LUSC	25340791		Figure 5
	RAB18	LUAD	25249344		Figure 1
hsa-miR-30d-5p	HOXA11	UCEC	25630974	2, 4	Figure 3
hsa-miR-320a	MYC	LIHC	28243124	1, 2, 4	Figure 2
	VDAC1	UCEC	26472185		Figure 4
hsa-miR-424-5p	WEE1	KIRC	23778472	1, 2, 4	Figure 4
hsa-miR-744-5p	TGFB1	KIRC/ KIRP	21991303	2, 4	Figures 9, 10

Validation Techniques: (1) Luciferase reporter assay, (2) quantitative RT-PCR, (3) Western blot analysis, (4) miRNA transfection.
